# Clinical Study of Labyrinthine Fistula in Cholesteatomatous Chronic Otitis Media: A Tertiary Care Hospital-Based Retrospective Study in a South Indian Population

**DOI:** 10.7759/cureus.42413

**Published:** 2023-07-25

**Authors:** Geetha Kishan Siddapur, Navneeta Gangwar, Manu Coimbatore Balakrishnan, Vandhana Murugesan

**Affiliations:** 1 Otolaryngology - Head and Neck Surgery, Sri Lakshmi Narayana Institute of Medical Sciences, Puducherry, IND; 2 Otolaryngology - Head and Neck Surgery, Jaipur National University Institute of Medical Sciences and Research Centre, Jaipur, IND

**Keywords:** foreign body, aural hygiene, risk factors, chronic otitis media, cholesteatoma, labyrinthine fistula

## Abstract

Introduction: Among the extracranial complications of cholesteatoma, the most common is labyrinthine fistula (LF). The causes are still poorly understood for cholesteatoma-induced labyrinthine fistula. Some of the possible factors described in the literature are the patient's age, duration of the disease, growth pattern of cholesteatoma, and disease aggressiveness. These affect the site of development of labyrinthine fistula. Cholesteatoma and its complications pose a great burden on the economic and health sector of developing nations.

Aim and objective: The objective is to estimate the incidence of labyrinthine fistula in cholesteatomatous chronic otitis media (COM) and analyze the clinical presentation and post-surgical improvement in hearing and vertigo in the study cohort.

Materials and method: The study was conducted in the Department of Otorhinolaryngology. It involved retrospective data collection of case records between 2018 and 2022. All patients diagnosed with chronic otitis media (COM) with cholesteatoma were reviewed retrospectively in a tertiary healthcare center. Of the 324 cases reviewed, 21 had an LF.

Results: The incidence rate of LF in our study was 6.48%. Sixteen (76.1%) patients were male, and five (23.9%) were female. The youngest patient was a 10-year-old male, and the oldest was a 51-year-old female. The mean ± standard deviation (SD) age was 34.09 ± 10.05 years. The left ear (76.1%) was affected more than the right ear. All cases were from rural areas, and 16 (76.1%) of them were farmers. Ear discharge (85%) was the most common symptom, followed by hearing loss (76%) and then vertigo (47%). A very peculiar risk factor of self-cleansing the ear was noticed in all patients. Out of the 21 patients who underwent surgery, it was observed that the lateral semicircular canal (LSCC) was the commonest site of the fistula. According to the Dornhoffer and Milewski classification, type II LF was the commonest type. In one patient with a type III LF, a foreign body (a piece of a twig) was found intraoperatively near the LSCC fistula site. Two patients had multiple fistulae. Six patients had associated mastocutaneous fistula, and one had facial nerve paralysis. All patients, except one, were free of vertigo following surgery. Postoperatively, the bone conduction thresholds were similar to the pre-surgical values in 12 of 16 (74%) patients.

Conclusion: The incidence of LF is still higher in developing countries, predominantly in rural populations, where the habit of self-cleansing the ear is a common practice. The common symptoms of COM with LF are ear discharge, hearing impairment, and vertigo. All the cases had a habit of frequent self-cleansing of the external ear as an important risk factor. Therefore, implementing awareness programs on maintaining aural hygiene in rural health centers may reduce the incidence of cholesteatomatous LF, thereby preserving hearing and vestibular functions and improving the quality of life. However, the above statement needs further validation with large multicenter studies.

## Introduction

Chronic otitis media (COM) squamous type, i.e., with cholesteatoma, leads to a variety of complications primarily due to its bone-eroding property. Among the extracranial complications of cholesteatoma, the most common is labyrinthine fistula (LF) [1]. The most common location of the LF is the lateral semicircular canal (LSCC) seen in 90% of cases [2]. The non-specific symptoms and signs of the disease make the diagnosis difficult [3]. A computed tomography (CT) scan is vital in aiding in the diagnosis of LF. The sensitivity of CT scans in diagnosing LF is 50%. However, with the advent of thinner cuts (0.5 mm slices) in high-resolution CT (HRCT), a sensitivity of 85%-100% has been reported [4,5]. Controversy remains in the treatment of LF due to cholesteatoma. A recent review of the literature proposes total removal of the matrix from the LF and restoration with bone sealing as the residual matrix can lead to progression of the cholesteatoma and increased bone resorption [6]. To avoid a second-look operation and maintain a dry, safe ear with meaningful hearing function, careful and total removal of the cholesteatoma matrix with the meticulous repair of the LF is required [7,8]. Cholesteatoma and its complications are highly prevalent in developing countries. Overcrowding due to poor socioeconomic status and lack of awareness of maintaining aural hygiene are known risk factors for the development of COM. However, few risk factors are not given much importance clinically. Those risk factors can be surprising and challenging to the operating surgeon in the intraoperative period [9].

We aim to estimate the incidence of labyrinthine fistula in cholesteatomatous chronic otitis media in our study population and analyze the clinical presentation and post-surgical improvement in hearing and vertigo in the study cohort.

## Materials and methods

The study was conducted after obtaining due approval from the Institutional Ethics Committee of Sri Lakshmi Narayana Institute of Medical Sciences, Pondicherry (affiliated to Bharat Institute of Higher Education and Research, Chennai) in 2022 with approval number IEC/C-P/12/2022.

In this retrospective observational study conducted between 2018 and 2022, a cohort comprising 324 cases of acquired cholesteatoma was analyzed, of which 21 cases were diagnosed with labyrinthine fistula.

Inclusion criteria

We included patients of both genders who presented to our department with acquired cholesteatoma during the study period from 2018 to 2022 and patients who underwent mastoid exploration for COM with cholesteatoma during the study period.

Exclusion criteria

Patients with a history of previous ear surgery and those with ear discharge but with isolated retraction pockets or isolated tympanic membrane perforation of pars tensa and flaccida without cholesteatoma were excluded.

Study procedure

Institutional Ethics Committee approval was obtained. Data regarding age, sex, clinical presentation, fistula test, audiological tests, radiological investigations, surgical procedure, and post-surgical improvement in hearing and vertigo were studied and tabulated. The surgical records of the patients were studied, and the diagnosis of LF was made based on CT and intraoperative findings. The LSCC type was defined according to the Dornhoffer and Milewski classification as follows [5]: type I, erosion of the bony labyrinth with an intact endosteum; type II, opened endosteum with an intact membranous labyrinth (type IIA, having intact perilymphatic space, and type IIB, having disturbed perilymphatic space either by ingrowth of cholesteatoma or by iatrogenic suctioning); and type III, opened perilymphatic space with involvement or destruction of the membranous labyrinth.

In the recent literature, it is one of the commonly used classification systems to classify findings of LF [5,8,9]. However, in this current study, we did not subclassify type II. In this retrospective analysis, a single-staged procedure in the form of canal wall down mastoidectomy and fistula repair with two layers of temporalis fascia was performed by the same surgical team.

Statistical analysis

Data were analyzed using Statistical Package for the Social Sciences (SPSS) version 20 (IBM SPSS Statistics, Armonk, NY, USA) and Microsoft Excel 2016 (Microsoft Corp., Redmond, WA, USA). To describe continuous data, means and standard deviations (SDs) were used. Frequencies and percentages were estimated for categorical data in our descriptive study.

## Results

**Figure 1:**
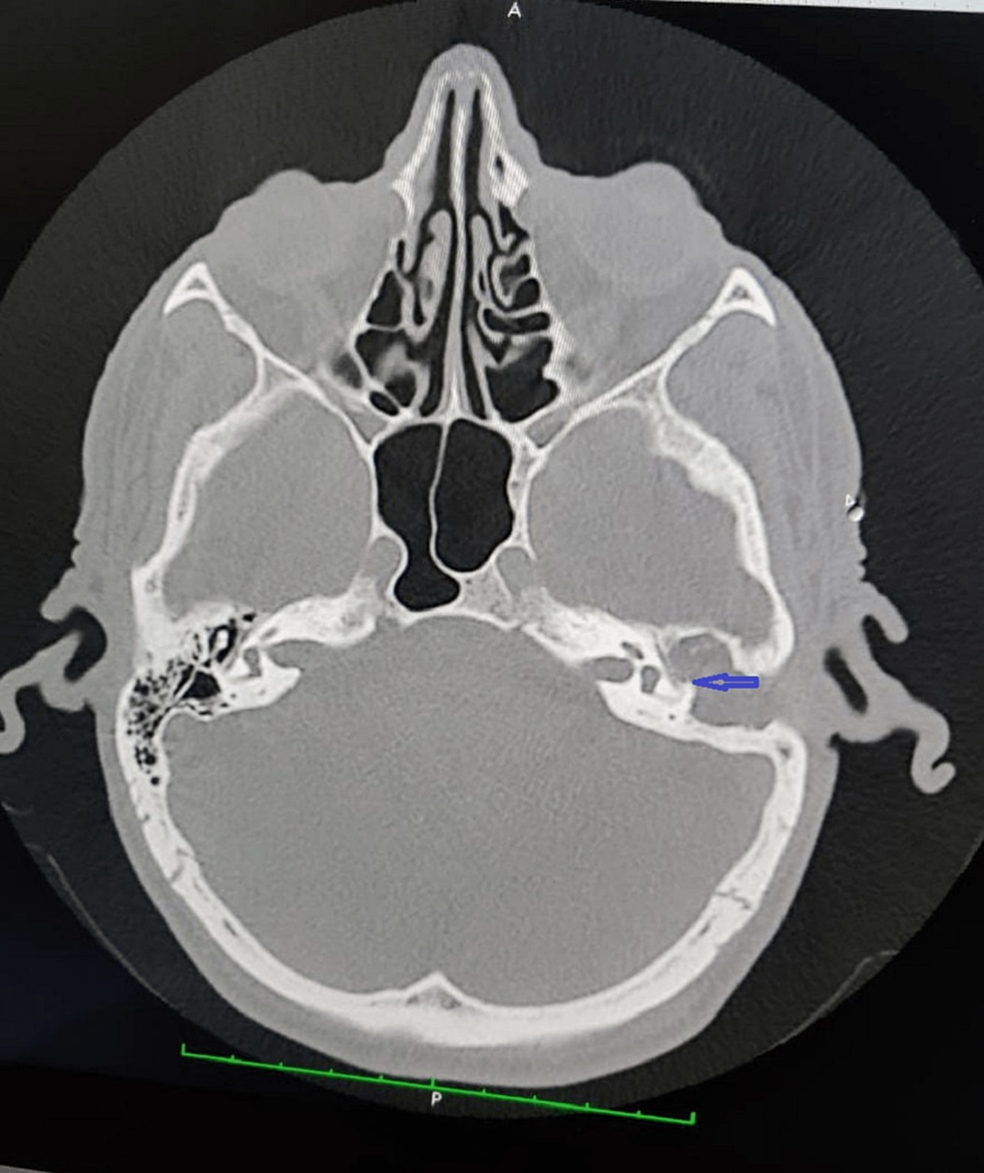
High-resolution computed tomography scan image Figure 1 depicts the axial section of a high-resolution computed tomography scan showing a type II labyrinthine fistula of the lateral semicircular canal (blue arrow).

In the present study, out of 324 cases of acquired cholesteatoma, 21 patients were found to have a labyrinthine fistula; the incidence was found to be 6.48%. The mean ± SD of age was 34.09 ± 10.05 years in our study population. It was more common in males (16, 76.1%) than in females (5, 23.9%). The left ear (76.1%) was affected more than the right ear. The largely affected population was from the rural sector of society (21 patients) with poor socioeconomic status. Most of them were farmers (16 patients). All the patients gave a peculiar history of self-manipulation of the external auditory canal for cleaning purposes. The most common symptoms were ear discharge in 18 (85%) patients and hearing impairment in 16 (76%) patients. Vertigo, aural fullness, headache, tinnitus, and otalgia were the other reported symptoms in our study population seen in 10 (47%), five (28.5%), four (19%), four (19%), and three (14%) patients, respectively. The fistula test was positive in three (14%) patients (Table 1, Figure 1).

**Table 1:** Degree of hearing loss in each fistula type in our study population Preoperatively, 24% (n = 5/21) of the patients had normal BC, 67% (n = 14/21) had preserved BC, and 9% (n = 2/21) had severe to profound SNHL. BC: bone conduction, SNHL: sensorineural hearing loss

Fistula type (N = 21)	Normal (<21 dB) (number (%))	Hearing level 21-40 dB (number (%))	Hearing level 41-70 dB (number (%))	Hearing level 71-90 dB (number (%))	Hearing level 90-110 dB (number (%))
I	4 (19%)	2 (9%)	2 (9%)	-	-
II	1 (5%)	6 (28%)	3 (14%)	1 (5%)	-
III	-	1 (5%)	-	-	1 (5%)
Total	5 (24%)	9 (41%)	5 (23%)	1 (5%)	1 (5%)

**Figure 2:**
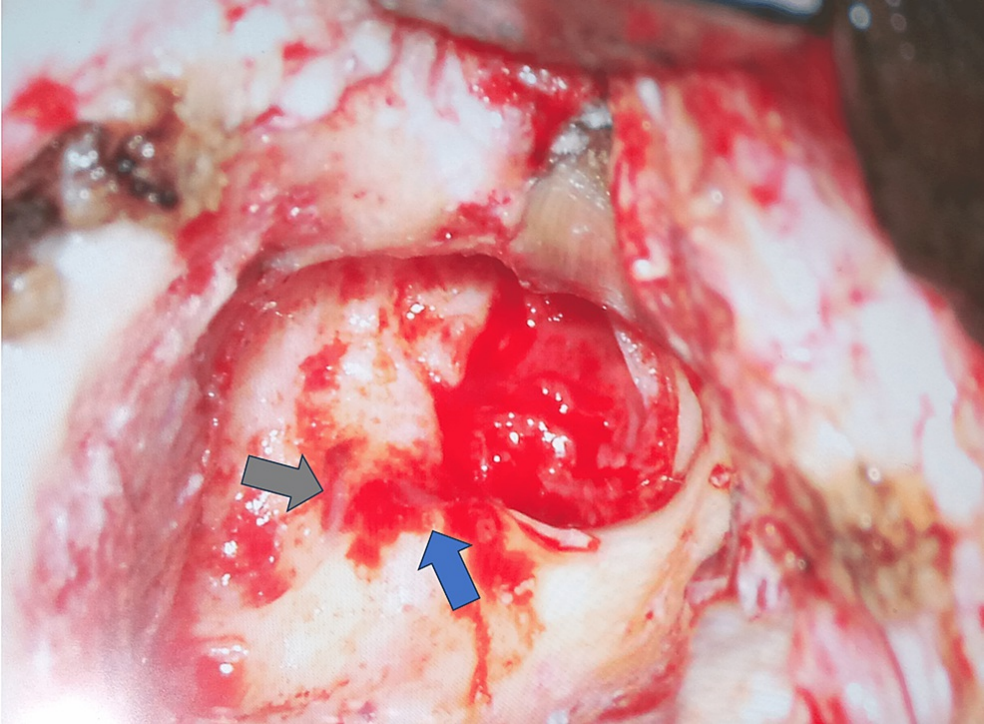
Intraoperative view of labyrinthine fistulae Figure 2 shows multiple fistulae (gray and blue arrows) in the lateral semicircular canal intraoperatively in the right temporal bone.

It was found that two patients had multiple fistulae intraoperatively (Figure 2). Six (29%) patients had associated mastocutaneous fistula, and one (4%) patient had associated facial nerve paralysis. Preoperatively, HRCT scans identified LF in 90% of the patients (Table 2).

**Table 2:** Preoperative high-resolution computed tomography scan predicting the presence of a fistula in the lateral semicircular canal Table 2 shows that in the present study, according to the Dornhoffer and Milewski classification, type II fistula was the commonest. Except for two patients, the fistula was diagnosed preoperatively on a computed tomography scan and confirmed during the operation, i.e., in 90% of the cases in our study.

Fistula type (N = 21)	Number (%)
Fistula type I	6/8 (75%)
Fistula type II	11/11 (100%)
Fistula type II	2/2 (100%)

**Figure 3:**
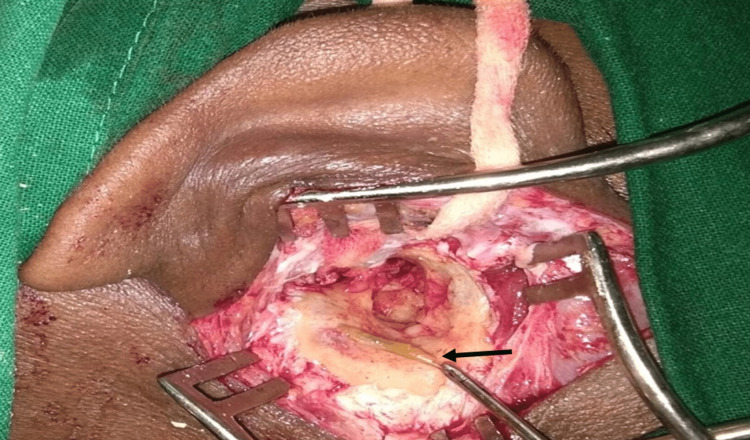
Intraoperative foreign body in a cholesteatoma patient Figure 3 shows a foreign body (a piece of a twig) (black arrow) found intraoperatively in the left mastoid cavity in a cholesteatoma patient.

In a patient with type III fistula, a foreign body (a piece of a twig) was incidentally discovered intraoperatively (Figure 3). The foreign body was present at the LSCC fistula site with one end of the foreign body inside the fistula and the other end pointing externally engulfed by the cholesteatoma. Of the 16 patients who came for follow-up, 15 (94%) were free of vertigo following surgery. Follow-up audiological tests were done at three and six months. Postoperatively, the bone conduction thresholds at six months were similar to the preoperative thresholds in 12 of 16 (74%) patients (Table 3).

**Table 3:** Postoperative bone conduction thresholds and fistula type Table 3 shows that hearing was improved or preserved in 74% of cases in our study. This rate is comparable to other current studies in which the cholesteatoma matrix was totally removed.

Hearing outcome (average bone conduction threshold) (N = 16)	Type I (number (%))	Type II (number (%))	Type III (number (%))	Total (number (%))
Improved/unchanged	6 (37%)	5 (31%)	1 (6%)	12 (74%)
Worse	-	1 (6%)	2 (12%)	3 (18%)
Dead	-	-	1 (6%)	1 (6%)

## Discussion

We encountered one such case where a foreign body (a piece of a twig) surprised us during routine mastoid surgery for cholesteatoma with a labyrinthine fistula. This case prompted us to retrospectively analyze all labyrinthine fistula patients with cholesteatomatous chronic otitis media. LF is a well-known complication of COM squamous type [10]. In the current study of four-year duration, LF in LSCC was found in 21 out of 324 patients operated for cholesteatoma, giving an incidence of 6.48%. Rosito et al. reported an incidence of 2.7% of LF in COM [8]. However, in the present study, the incidence is relatively higher, which may be attributed to the short study period of four years compared to other studies reporting the incidence for a study period of a minimum of five years. Another study reported an incidence rate of 4%-12% of labyrinthine fistula in COM [11]. The most affected patients were found to be from rural areas with poor socioeconomic status. LF, which is acquired following cholesteatoma as a complication of chronic otitis media, may not manifest straightforwardly. A combination of otologic symptoms may result in complex ways that are difficult for the patient to explain, especially when there is a lack of awareness among people. Hence, rural people with inadequate access to information and healthcare settings tend to report cholesteatoma late, which then results in the formation of a labyrinthine fistula [12].

A history of vertigo can be seen in 64% of patients with LF, with a positive fistula test in 50% of patients, and in 15% of patients, profound sensorineural hearing loss (SNHL) can be seen among patients with LF due to cholesteatoma in general [13]. In our study, vertigo was seen in 47% of cases, with a positive fistula test in 14% of cases, and 9% of cases had severe to profound hearing loss.

The commonest site for LF due to cholesteatoma was found to be the lateral semicircular canal, which can be due to its anatomic proximity to the middle ear and mastoid [13]. This finding was similar to our study. In a study by Magliulo et al., 10 (47%) cases had absent clinical symptoms and signs suggestive of vestibular pathology, despite the bone-eroding disease in the labyrinth [14]. This can be possibly explained by the intact membranous labyrinth and protection by the cholesteatoma matrix of the inner ear fluid from sound waves or middle ear pressure [15]. Many patients will have normal hearing unless there is movement of toxins or inflammatory mediators into the fluid of the inner ear. The presence of membrane limitans (utricular-endolymphatic valve) and the perilymphatic system's connective tissue that gives relative independence from the labyrinthine fluid of the posterior labyrinth to that of the cochlea are some of the other mechanisms that are proposed in the literature for the absence of vestibular symptoms in LF [16].

Data regarding other clinical features have poor reliability due to it being affected by variables such as the patient's memory and avoidance of prompt medical care. Vertigo was most commonly reported, which was because most of the patients were farmers and dizziness/vertigo decreased their work efficiency [17]. Hearing loss will usually be a complaint of long duration, which suggests that it may be the most neglected symptom [18]. The risk factors that were identified in the present study included self-manipulation for cleaning purposes as well as occupational exposure to dust and vegetative flora as most of them were farmers. Some of the causes of secondary acquired cholesteatoma include infection, trauma, or impaction of skin into the middle ear, which can occur as a consequence of self-cleaning of the external auditory canal as seen in our cases. Hence, the lack of awareness regarding aural hygiene and the odd practice of self-cleaning is seen to be associated with an increased incidence [18].

Using the Dornhoffer and Milewski classification, 16 (38%) cases were diagnosed as type I fistula, 22 (52%) as type II fistula, and four (10%) as type III fistula in a study by Meyer et al. [2]. Gocea et al. reported similar findings [19]. Our study also had type II fistula as the commonest type. In the present study, a foreign body was found intraoperatively in a patient with type III labyrinthine fistula. It was present at the LSCC fistula site with one end of the foreign body inside the fistula and the other end pointing externally engulfed by the cholesteatoma. No similar finding has been described in the literature. This provides curious insight into how self-cleansing with twigs could be a potential risk factor. However, further studies with large sample sizes are required to support the evidence.

In our study, we achieved hearing improvement/non-worsening in 74% of cases of COM with labyrinthine fistula after surgery. Meyer et al. reported hearing improvement in 75% of COM with labyrinthine fistula cases after surgery [2], whereas Sagar et al. reported an improvement/non-worsening of hearing thresholds in 92% of cases of COM with labyrinthine fistula after surgery [11]. The surgical management of cholesteatoma-induced LF has some controversies that mostly revolve around the matrix removal covering the fistula and lowering of the posterior canal wall. Over the decades, the concepts are evolving, and recently, there has been a shift of consensus toward the complete removal of the cholesteatoma matrix from the fistula [20]. The residual cholesteatoma epithelium lining the fistula can produce collagenase and result in labyrinthitis in the long term. This risk is more compared to the possible labyrinthine damage due to the removal of the residual cholesteatoma epithelium lining the fistula as shown in studies [21]. Kobayashi et al. reviewed 23 patients with fistula and found that the size, number, and location of the fistulae and the preservation of the posterior canal wall did not have any statistically significant impact on patients' hearing [22]. Chen et al. have shown that to avoid a second-look operation and maintain a dry, safe ear, careful and total removal of the cholesteatoma matrix with the meticulous repair of the LF is required. It also provides a significant hearing function [23,24].

The strength of the study is the identification of a possible underreported risk factor in our study population. The limitations of the study are the retrospective design and the relatively small sample size of labyrinthine fistula patients (N = 21), although the total number of patients with acquired cholesteatoma studied was 324, which is comparable to other studies [2,19].

## Conclusions

Ear discharge, hearing impairment, and vertigo were the common symptoms of labyrinthine fistula due to cholesteatoma in our study population. Among the patients who came for follow-up, 74% of them had preserved hearing, and 94% of patients were free of vertigo postoperatively. It is ascertained from the present study that the incidence of labyrinthine fistula due to an acquired cholesteatoma is relatively higher in developing countries with a predominantly rural population, where the habit of self-cleansing the ear is a common practice. However, the above statements need further validation with large multicenter studies. It is also observed that the left ear is affected more and that labyrinthine fistula is more common in males. Implementing awareness programs on maintaining aural hygiene in rural health centers may reduce the incidence of cholesteatomatous labyrinthine fistula, thereby preserving hearing and vestibular functions and improving the quality of life.
